# Effects of storage time and temperature on the antimony and some trace element release from polyethylene terephthalate (PET) into the bottled drinking water

**DOI:** 10.1186/s40201-014-0133-3

**Published:** 2014-11-13

**Authors:** Ebrahim Molaee Aghaee, Mahmood Alimohammadi, Ramin Nabizadeh, Gholamreza Jahed khaniki, Simin Naseri, Amir Hossein Mahvi, Kamyar Yaghmaeian, Hassan Aslani, Shahrokh Nazmara, Babak Mahmoudi, Maryam Ghani

**Affiliations:** Department of Food Hygiene and Control, Faculty of Veterinary Medicine, Tehran University, Tehran, Iran; Department of Environmental Health Engineering, School of Public Health, Tehran University of Medical Sciences, Tehran, Iran; Center for Water Quality Research (CWQR), Institute for Environmental Research (IER), Tehran University of Medical Sciences, Tehran, Iran

**Keywords:** PET, Bottled water, Antimony, Trace elements

## Abstract

**Background and objectives:**

Heavy metals are considered as one of the major contaminants that can enter into the bottled waters. Antimony (Sb) is a contaminant, which may leach from the polyethylene terephthalate (PET) bottles into the water. The aim of this study was to investigate the content of antimony and other trace elements in bottled waters which was kept in varied storage conditions and temperatures.

**Materials and methods:**

Five time-temperature treatments were carried out on five different brands of commercially available bottled waters. Heavy metal measurement was performed by Inductively Coupled Plasma-Atomic Emission Spectroscopy (ICP-AES) method. Analysis of the collected data was processed by SPSS software.

**Results:**

Antimony concentration was the main concern in our study. The concentrations increased in each of the sample during storage period at all temperatures. The results for different conditions were as follow: at 40°C, in outdoor and at room temperature the Sb concentrations were below the MCLs, i e. 6 ppb. However, at 65°C and 80°C for longer storage times Sb concentration exceeded the MCLs, and variations between the samples were significant (p ≤ 0.05). Storage time and temperature effects on the content of some other trace elements such as Al, Fe were also significant (p ≤ 0.05).

**Conclusion:**

By increasing the duration of storage time and temperatures, antimony leaching from the PET bottles into the bottled water increased. The concentration of Al demonstrated an increase in higher temperatures and storage duration, whereas the content of Fe demonstrated no significant differences.

## Introduction

Consumption of bottled water has become increasingly popular worldwide. Population growth and lack of access to appropriate drinking water has also increased use of bottled waters; and in some areas, some major concerns to the consumers are environmental pollutants and its adverse effects on the local water supply, as well as, a number of the local municipality water irregularities, such as off odor and taste of the water, high amount of fluoride and chloride. On the other hand, people with immunity system deficiency require more safe drinking water. Moreover, it is believed that bottled water is safer and has higher quality than other water resources which increased its consumption [1–3]. Hence, higher standards and quality control measures are required on the safety, chemical and microbial properties of the bottled waters. According to the World Health Organization (WHO), over 1.8 million people, particularly children, die annually due to water borne diseases, which have been, and is one of the leading and still prevalent mortality causes worldwide [4,5].

Antimony, titanium or germanium compounds are used as catalysts in the manufacturing of polyethylene terephthalate (PET) bottles, where polymerization of terephthalic acid monomers and ethylene glycol occurs. Among these, Antimony trioxide is one of the main catalysts used, and the amount of Antimony trioxide found in the bottles varies between 100-300 mg/kg [6]. Antimony is regarded as one of the major drinking water contaminants, which exceeds the maximum contaminant level (MCL), i.e. 6 ppb, under a number of conditions. Short term exposures to the levels higher than MCL, can cause side effects such as nausea, vomiting and diarrhea. Higher blood cholesterol and lower blood sugar are other side effects when exposed for a longer period of time [7]. According to Shotyk and Krachler high storage temperatures and sunlight irradiation could increase the Sb release into the bottled water [8].

Epidemiological studies revealed a significant link between diseases and the presence of trace toxic elements such as As, Cd, Pb and Sb in bottled waters, as well as the high amounts of essential micronutrients such as Zn, Se and Co. Although, some trace elements such as Cr, Co and Se are essential for human life, these mineral if consumed in higher rates than the suggested required daily(RD) amounts can be harmful [9,10].

Due to a considerable increase in the PET bottled water consumption worldwide, the purpose of this study was to investigate the trace element contents in bottled waters and the conditions, which affect the release of Sb and/or other trace elements into the water.

## Materials and methods

Five different popular brands of bottled water with the highest rate of consumption were purchased from the local grocery markets, in Tehran, Iran. The main criteria for selecting the brands in this study, was their production in different regions of Iran and different environments. The five storage conditions designed for the investigation of the samples were, storage at room temperature (25 ± 2.6°C), outdoors and sunlight (31 ± 4.6°C) and 40°C and the experiment intervals were defined as follows, weeks 0 (immediately after receiving), 1, 2, 4, 6 and 8 were performed. Storage at 65°C, and the experiments was done in days zero (immediately after receiving), 3, 7 and weeks 2, 4 and 6. Storage at 80°C at test days zero (immediately after receiving), 1, 2, 3, 4 and 7 was considered for the other storage condition. In the defined intervals for each temperature condition, 0.5 L samples were acidified with 1 mL 65% nitric acid to prevent sedimentation and then were digested and placed on an electrical heater to obtain a concentrated volume of 25 mL. Furthermore, in order to detect the very low trace amounts of the elements, the condensation process was carried out at a very low temperature over a long period of time to prevent boiling of the samples. For short-term storage, i e. before sample injection to ICP-AES (model Spectro ARCOS.), and in order to prevent any variation in trace elements concentrations pH of the samples were lowered to 2 by using a pure 65% nitric acid. Chemical examinations were performed by Ion Chromatography (IC) for anions and cations, additional procedures were performed according to the Standard Methods for the Examination of Water & Wastewater (APHA 21st Ed.). Statistical analyses was performed using the SPSS (11.5) software. A probability level of *p* < 0.05 was considered statistically significant.

## Results

Chemical properties of all five brands of the different bottled waters were measured for a better consideration of the examined samples. Both labeled data and analyzed characteristics are presented in Table 1. All samples pH were in the range from 7.2 to 7.9 which specified by the labels as well as measurements.

**Table 1:** **Physicochemical properties of bottled water samples**

**Brand no.**	**F**	**Cl**	**NO**_**2**_	**NO**_**3**_	**SO**_**4**_	**Na**	**K**	**Ca**	**Mg**	**pH**
1	0.07 ± 0.03	1.77 ± 0.04	0	4.02 ± 0.14	9.26 ± 0.06	3.9 ± 0.16	0.21 ± 0.007	42.7 ± 19.37	3.99 ± 0.25	7.42 ± 0.06
Label	0.2	6	0	7	10	5	1	29.6	4	7.3
2	0.27 ± 0.007	8.75 ± 0.02	0	14.4 ± 0.42	26.25 ± 0.21	8.44 ± 0.0	0.82 ± 0.17	39.45 ± 36.5	18.85 ± 0.35	7.59 ± 0.09
Label	0.23	16.4	0	14	21	10.8	1.37	62.7	20.3	7.5
3	0.07 ± 0.02	1.09 ± 0.03	0	3.95 ± 0.23	6.32 ± 0.1	0.98 ± 0.12	0.31 ± 0.03	31.95 ± 2.47	7.39 ± 0.53	7.67 ± 0.05
Label	0.07	6	0	7	3	1	0.1	32.1	7.61	7.6
4	0.12 ± 0.007	0.64 ± 0.02	0	2.44 ± 0.21	20.35 ± 0.21	4.02 ± 0.05	1.59 ± 0.01	0.01 ± 0.007	0.0 ± 0.007	7.29 ± 0.07
Label	0.11	0.7	0	2.3	19	4.7	1.9	9.8	2.3	7.2
5	0.15 ± 0.01	4.27 ± 0.1	0.06	12 ± 0.14	24.85 ± 0.07	10.7 ± 0.14	0.7 ± 0.02	106.5 ± 21.92	19.5 ± 0.56	7.42 ± 0.07
Label	0.2	6.1	0	7.5	10.6	10.5	0.6	56.4	15.4	7.4
EPA,2002	2	250^a^	3.3	44	250^a^	-	-	-	-	6.5-8.5^a^

^a^Secondary Maximum Contaminant Level (SMCL).

Content of trace metals in the bottled water samples at the initial stage of this study are illustrated in Table 2.

**Table 2:** **Trace metals content of bottled water samples**

**Metal/Brand**	**Unit**	**1**	**2**	**3**	**4**	**5**	**EPA (2002)**
Al	ppb	3.82 ± 0.03	6.17 ± 0.45	12.77 ± 0.74	7.52 ± 1.23	6.67 ± 0.24	200^b^
As	ppb	N.D^a^	N.D	N.D	3.37 ± 1.02	N.D	10
B	ppb	14.17 ± 0.95	25.97 ± 1.09	10.4 ± 1.48	35.52 ± 2.93	23.5 ± 1.76	-
Be	ppb	N.D	N.D	N.D	N.D	N.D	4
Ba	ppm	N.D	25.02 ± 2.08	10.37 ± 1.23	31.35 ± 4.10	31.47 ± 2.86	2
Cd	ppb	N.D	N.D	N.D	N.D	N.D	5
Co	ppb	N.D	N.D	N.D	N.D	N.D	-
Cr	ppb	0.17 ± 0.03	0.17 ± 0.09	N.D	0.20 ± 0.07	0.19 ± 0.05	100
Cu	ppb	2.25 ± 0.56	4.27 ± 1.16	2.07 ± 0.53	3.4 ± 0.77	7.92 ± 0.88	1^b^-1.3ppm^c^
Fe	ppb	1.57 ± 0.38	1.55 ± 0.42	2.77 ± 0.53	2.4 ± 0.63	5.77 ± 0.74	300^b^
Hg	ppb	N.D	N.D	N.D	N.D	N.D	2
Li	ppb	N.D	2.15 ± 0.56	0.57 ± 0.24	2.95 ± 0.42	0.62 ± 0.17	-
Mn	ppb	N.D	N.D	N.D	N.D	N.D	5^b^
Mo	ppb	N.D	N.D	N.D	N.D	N.D	-
Ni	ppb	1.5 ± 0.35	1.17 ± 0.38	0.37 ± 0.10	5.72 ± 1.52	1.47 ± 0.38	100
P	ppb	2.1 ± 0.35	1.9 ± 0.63	1.6 ± 0.35	1.85 ± 0.35	1.9 ± 0.28	-
Pb	ppb	0.35 ± 0.07	N.D	N.D	0.75 ± 0.14	N.D	10
Sb	ppb	0.64 ± 0.05	0.68 ± 0.09	1.41 ± 0.12	1.85 ± 0.19	0.44 ± 0.04	6
Se	ppb	0.36 ± 0.07	0.47 ± 0.10	0.35 ± 0.07	0.38 ± 0.04	0.40 ± 0.14	5
Sn	ppb	0.22 ± 0.03	0.27 ± 0.10	0.30 ± 0.21	0.97 ± 0.38	0.48 ± 0.09	-
Sr	ppb	28.35 ± 1.62	49.77 ± 1.44	47.62 ± 1.59	36.22 ± 2.51	49.65 ± 4.45	-
Ti	ppb	N.D	N.D	N.D	N.D	N.D	-
V	ppb	0.42 ± 0.10	0.53 ± 0.16	N.D	2.55 ± 0.98	N.D	-
Zn	ppb	36.15 ± 6.57	3.30 ± 0.77	2.12 ± 0.88	25.62 ± 2.86	9.37 ± 1.30	5ppm^b^

^a^Not Detected.

^b^Secondary Maximum Contaminant Level (SMCL).

^c^Action Level.

**Figure 1:**
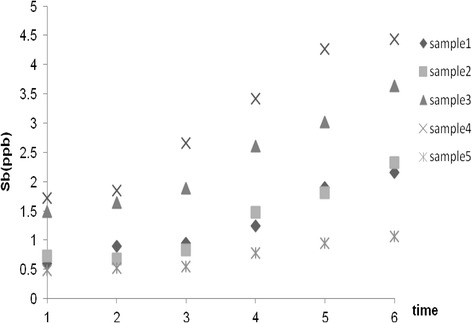
**Antimony concentration changes during storage (outdoor, room and 40°C** **).**

Antimony concentrations, as the main trace element of the study, in all the samples at the day zero and at the initial stages of the examinations were below the MCL (6 ppb), but the levels were higher for samples 3 and 4 than others samples (p ≤ 0.05). Under the outdoor and sunlight condition with the average temperature of 31 ± 4.1°C, the concentration of Sb increased in all samples over time. At room temperature storage (25 ± 2.6°C) as well as in 40°C condition, the trend of changes were similar to the outdoor conditions and variations were insignificant, in both cases (p > 0.05). The Sb content of bottled waters was still lower than the MCL at the end of the study (after 8 weeks) for the outdoor storage, the interior storage and 40°C storage conditions. Our findings demonstrated that there was a high correlation between the Sb initial concentrations and its amounts during the study. By considering the fact that Sb concentration changes was similar at the three storage conditions and the trends were the same Figure 1 demonstrates the average Sb changes for the storage duration in the three above mentioned conditions.

**Figure 2:**
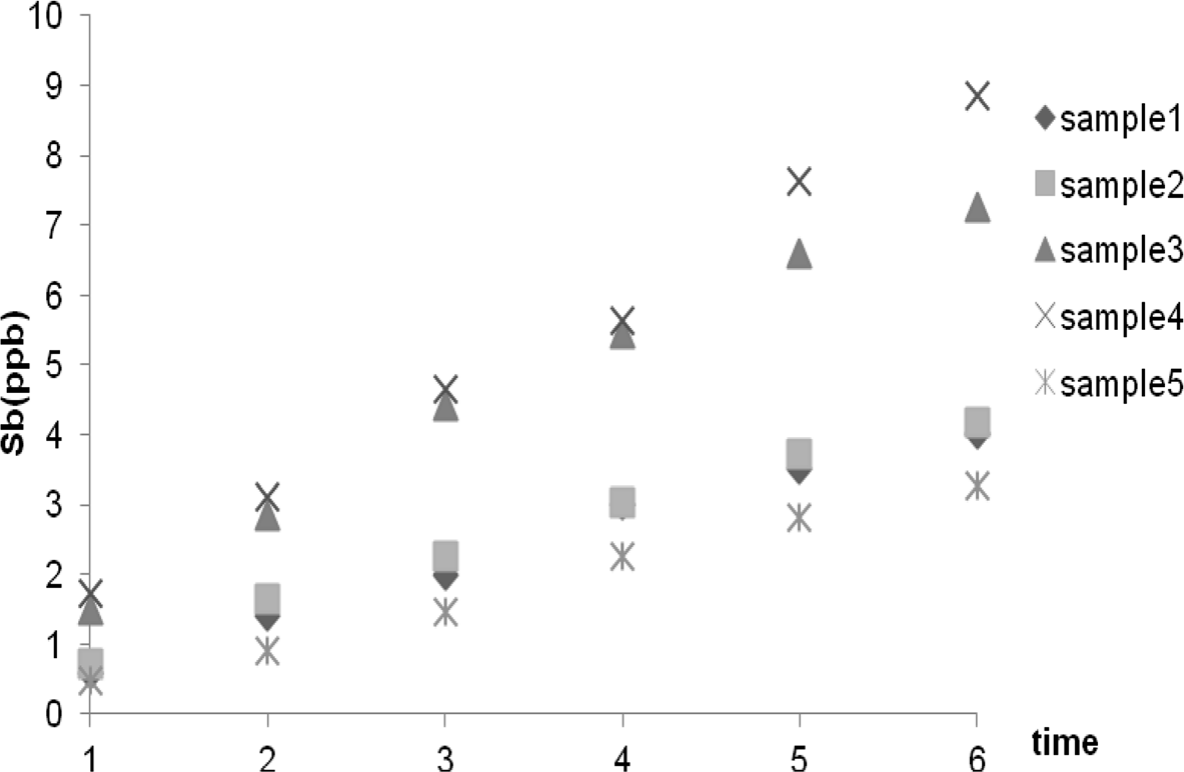
**Sb average concentration changes during storage (65°C** **).**

Antimony concentrations at 65°C increased over the MCL following the two weeks storage period for samples 3 and 4, whereas in the case of other samples it was still lower than the MCL by the end of the study (Figure 2).

**Figure 3:**
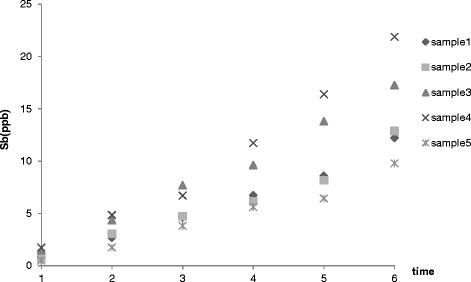
**Sb average concentration changes during storage (80°C** **).**

In addition, as illustrated in Figure 3, at 80°C, Sb concentration increased over the MCL in the second day of the storage time for samples 3 and 4, while for the other samples it was observed in the day three.

**Figure 4:**
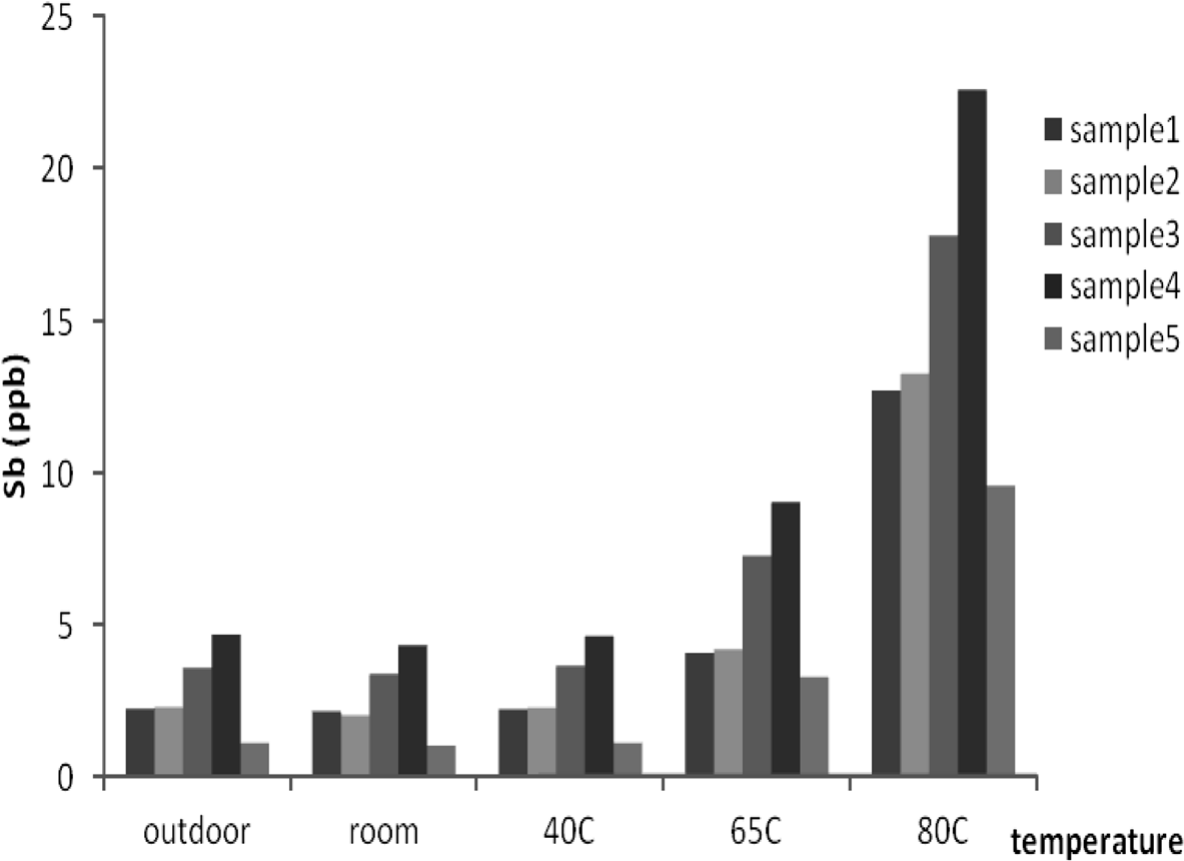
**Concentration of Sb in the samples at the end of each storage condition.**

At the end of the study, the highest concentration for Sb was recorded at 80°C storage conditions, and as can be seen in Figure 4 the lowest concentration was observed for outdoor storage room and at the 40°C storage temperature.

Storage time and temperature effects on the Al concentrations were significant, particularly for the samples stored at 65°C and 80°C (p ≤ 0.05) which demonstrated a relevant increase during storage time. In case for Fe concentration the storage time and temperature effect was significant, mainly for the samples stored at 80°C (p ≤ 0.05), and demonstrated a relevant decrease during storage time. Other trace metals measured in this study included, As, B, Ba, Cr, Cu, Ni, Pb, Se, Sn, Sst, Va and Zn. These elements showed no statistically significant differences (p > 0.05) for storage conditions. The elements B, Cr, Cu, Ni, Se, Sn, St and Zn displayed significant variations (p ≤ 0.05) in each time zone and each temperature storage condition. B, Cr, Cu and Sn concentrations decreased in their time zones in all five temperature storage conditions, while Ni and St demonstrated a slightly increase in their time zone in each temperature storage condition. Selenium and Zn contents displayed both an increasing and decreasing trend by the storage time and at the different temperatures.

The elements Be, Cd, Co, Hg, Mn, Mo and Ti concentrations were below detection limit at all the storage conditions.

## Discussion

Antimony and other trace element concentrations in the samples that were measured at the beginning of the study were all below the promulgated standards, except for Barium. Higher temperatures and longer storage times had a substantial effect (p ≤ 0.05) on the increase of Sb and Al concentrations, while, a decrease was observed in case for Fe.

Westerhoff et al. [7] showed that high storage temperatures and sunlight exposure increased the leakage of antimony into the bottled waters. Antimony concentration in samples was 0.095 - 0.521 ppb which was lower than that MCL recommended by the United States Environmental Agency (USEPA). The initial mean concentrations were 0.195 ± 0.116 ppb, and after 3 monthas period at 22°C rose to 0.226 ± 0.160 ppb. The amounts of Sb exceeded the MCL in the following conditions: 60°C in 176 days, 65°C in 38 days, 70°C in 12 days, 75°C in 4.7 days, 80°C in 2.3 days and 85°C in 1.3 days [7].

In this study, the average Sb concentrations were approximately 1.01 ± 0.59 ppb at the initial stage of the experiments, and by the end of the study (after 8 weeks) at room temperature (25 ± 2.6°C) rose to 2.54 ± 1.58 ppb. In case of 65°C, samples 3 and 4 which had the highest initial Sb content, after a two week periods exceeded the MCLs. while the other samples remained below the MCL. Whereas, as the temperature rose to 80°C after two days the Sb amounts exceeded the MCL for samples 3 and 4, while for the other samples it was observed after day three.

Various experiments by Xiaoliang Cheng et al. [6] demonstrated that 16 trace elements including Sb were leaked into the bottled waters tested in the following conditions, cooling with ice-cold water, warming by boiling, microwave, incubation at low pH, outdoor with sunlight irradiation and storage inside the automobiles. The study established that warming by boiling and microwave could considerably augment the leaching process of Sb into water and in some cases exceeded the MCL levels. However, both incubation at low pH and also cooling with ice-cold water, revealed no significant effects; while the outdoor with sunlight irradiation and sunlight exposure, and the storage inside automobiles increased the leakage, but still was below the required MCLs [6].

The results revealed that minor leakage of Sb from PET bottles was due to plastic surface pollution at some stages of manufacturing process; while any major leakage was due to the changes in the bottle’s storage conditions. The effect of various storage conditions on the leakage of Sb in the remaining 15 samples were not considerable and/or far below the recommended MCLs in all samples. Conversely, in this study, an increase in Al concentration was observed.

According to Shotyk et al. [11] the comparison between the natural aggregates of Sb in the underground waters versus the PET bottled waters (same water before and after bottling) indicated Sb leakage from these bottles. It was established that Sb content of the bottled was 30 times higher than the water in the glass container [11]. Keresztes et al. [12] studied ten different brands of pure and sparkling waters. The study demonstrated that the content of Sb in the sampled bottles was 0.03-0.1 ppt, which increased at room temperature, and in darkness. The study also revealed that light and temperature exposures could raise the concentration levels of Sb [12].

A study performed by Guler et al. [13] demonstrated that the levels of Sb in the various samples were higher than the Turkish legislative limits; 24% of natural spring water samples (24 out of 100), 28.6% of natural mineral water samples (2 out of 7) and 54.4% of drinking water samples (6 out of 11). The present study, analyzed Ba concentration at the initial stage of the study in 4 samples, as illustrated in Table 2, the results were higher than USEPA recommended MCL level [13].

The concentrations of Sb’s in 132 brands from 28 countries were studied by Shotyk et al. [8]. Two of the brands had higher values than the MCLs of Japan (2 μg/l) which was mainly due to the use of Sb_2_O_3_ as the catalyst in the PET process. In 14 brands of bottled waters in Canada, Sb content during a 6 month storage period at room temperature increased approximately 19%, while the 48 brands of 11 European countries demonstrated an increase of 90% under the same condition. The analysis of the underground spring water contained 1.7 ± 0.4 ng/L Sb, following 6 month period of storage, the Canadian bottles Sb levels rose to 26.6 ± 2.3 ng/L, while the German bottled water demonstrated 28.1 ± 3.8 ng/L [8].

In a research conducted by Baba et al. [14], the Co content levels were 7-11 ppb in sampled bottles, and the Sb levels reported were lower than 1 ppb, which were below the detection limit. However, it should be mentioned that to date, WHO, EPA and Turkish standards have defined no standard for Co levels. Moreover, in the present study, the concentration of Co levels, in all samples were below the limit of detection (LOD) [14].

## Conclusion

The study has concluded that Antimony (Sb) may leach from the polyethylene terephthalate (PET) bottle into the bottled water. This particularly can be observed in water bottles which are stored at high temperatures for long periods. Furthermore, other trace elements should not be a concern, since these trace element concentrations with the exception of Ba, were far below the recommended MCLs; also, during the storage period under different conditions the concentrations remained lower than the permitted levels. Hence, this study has concluded that to prevent the increase of Sb in bottled waters, it is strongly recommended that commercial markets not store the bottled waters at high temperatures for long periods, especially in summertime. Last of all, the consumer should not leave the bottled waters inside their vehicles’ and/or closed areas due to the high temperatures which occur in these conditions.
